# Uliginosin B, a Possible New Analgesic Drug, Acts by Modulating the Adenosinergic System

**DOI:** 10.1155/2016/5890590

**Published:** 2016-03-21

**Authors:** Eveline Dischkaln Stolz, Paola Fontoura da Costa, Liciane Fernandes Medeiros, Andressa Souza, Ana Maria Oliveira Battastini, Gilsane Lino von Poser, Carla Bonan, Iraci L. S. Torres, Stela Maris Kuze Rates

**Affiliations:** ^1^Programa de Pós-Graduação em Ciências Farmacêuticas, Universidade Federal do Rio Grande do Sul, 90610-000 Porto Alegre, Brazil; ^2^Laboratório de Farmacologia da Dor e Neuromodulação: Investigações Pré-Clínicas, Departamento de Farmacologia, Instituto de Ciências Básicas da Saúde, Universidade Federal do Rio Grande do Sul, 90046-900 Porto Alegre, Brazil; ^3^Departamento de Bioquímica, Instituto de Ciências Básicas da Saúde, Universidade Federal do Rio Grande do Sul, 90035-003 Porto Alegre, Brazil; ^4^Laboratório de Neuroquímica e Psicofarmacologia, Faculdade de Biociências, Pontificia Universidade Católica do Rio Grande do Sul, 90619-900 Porto Alegre, Brazil

## Abstract

Uliginosin B (ULI) is a natural acylphloroglucinol that has been proposed as a new molecular scaffold for developing analgesic and antidepressant drugs. Its effects seem to be due to its ability to increase monoamines in the synaptic cleft by inhibiting their neuronal uptake without binding to their respective transporters, but its exact mode of action is still unknown. Considering the importance of the purinergic system to pain transmission and its modulation by monoamines availability, the aim of this study was to investigate the involvement of adenosinergic signaling in antinociceptive effect of uliginosin B. The selective adenosine A_1_ receptor antagonist DPCPX and the selective A_2A_ antagonist ZM 241385 prevented the effect of ULI in the hot-plate test in mice. Pretreatment with inhibitors of adenosine reuptake (dipyridamole) or adenosine deaminase (EHNA) did not affect the ULI effect. On the other hand, its effect was completely prevented by an inhibitor of ecto-5′-nucleotidase (AMPCP). This finding was confirmed* ex vivo*, whereby ULI treatment increased AMP and ATP hydrolysis in spinal cord and cerebral cortex synaptosomes, respectively. Altogether, these data indicate that activation of A_1_ and A_2A_ receptors and the modulation of ecto-5′-nucleotidase activity contribute to the antinociceptive effect of ULI.

## 1. Introduction

Uliginosin B (ULI) is a dimeric acylphloroglucinol consisting of filicinic acid and phloroglucinol moieties, which occurs in* Hypericum* species native to South America [1]. This molecular pattern has been proposed as a prototype to develop analgesic and antidepressant drugs [2–5].

Preclinical studies suggested that ULI has antidepressant properties, which seems to be due to its ability to increase monoamines availability in the synaptic cleft by inhibiting their neuronal uptake [2]. Nevertheless, ULI does not bind to the monoamine sites on neuronal transporters, which indicates that it acts differently from the classical antidepressants [2]. It is noteworthy that ULI deserves attention as a drug potentially useful to reduce the dose of morphine in clinical practice [6]. Its antinociceptive effect involves the activation of monoaminergic, glutamatergic, and opioid receptors, apparently without binding to these receptors [2, 3, 5]. Therefore, other molecular targets for ULI might be considered.

The relationship between purinergic system/nociceptive pathways has been reported [7]; numerous studies described the interaction between purinergic, monoaminergic, and opioid pathways [8–14].

Adenosine triphosphate (ATP) stimulates cellular excitability, augments the release of excitatory amino acids, initiates a nociceptive response, and can lead to apoptosis [15, 16]. ATP released from cells into the extracellular space has a short half-life in the extracellular milieu since it is rapidly degraded to adenosine diphosphate (ADP), adenosine monophosphate (AMP), and adenosine by ectonucleotidase pathway, which includes the E-NTPDase family (ectonucleoside triphosphate diphosphohydrolase) and ecto-5′-nucleotidase (for review see Robson et al. [17] and Zylka [18]). These enzymes control the availability of ligands (ATP, ADP, AMP, and adenosine) to activate purinoceptors, as well as the duration of receptor activation. In addition, these enzymes may provide a protective function by maintaining extracellular ATP/ADP and adenosine levels within physiological concentrations (for review see Burnstock [19]). Adenosine levels are also controlled by deamination to inosine through adenosine deaminase (ADA), cell release, and reuptake through nucleoside transporters (NTs) in bidirectional equilibrative processes driven by chemical gradients and unidirectional concentrative processes driven by sodium electrochemical gradients [20, 21]. The activation of adenosine receptors appears to be involved in the modulation of nociceptive and inflammatory pathways [7]. These effects depend on the availability of adenosine in the synaptic cleft, as well as intensity and modality of the stimulus [11].

Interestingly, several drugs that increase monoamine availability or act through the activation of opioid receptors present antinociceptive effect mediated by activation of adenosine receptors [12–14].

In view of these observations, the aim of this study was to investigate the involvement of purinergic pathway in the antinociceptive effect of ULI, including the effect of ULI on adenosine metabolism.

## 2. Material and Methods

### 2.1. Uliginosin B Obtention

**Figure 1:**
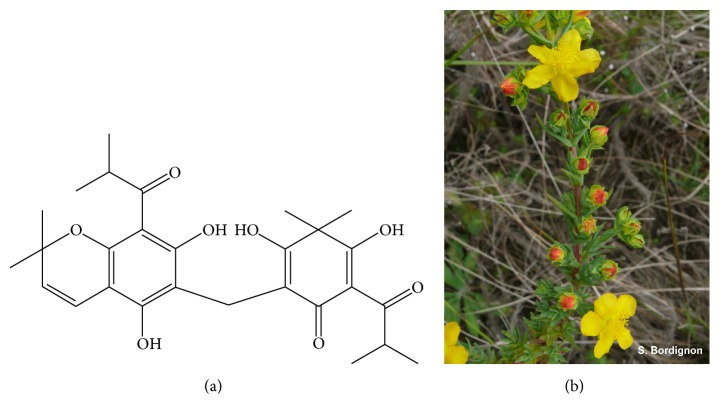
Uliginosin B structure (a).* Hypericum polyanthemum*, plant used to obtain ULI (b).

ULI (Figure 1(a)) was obtained according to Stolz and coworkers [3] from* n*-hexane extract of the aerial parts (all sections above ground) of* Hypericum polyanthemum* Klotzsch ex Reichardt (Hypericaceae) (Figure 1(b)), harvested in Caçapava do Sul, Brazil (voucher specimen ICN 175915). Plant collection was authorized by the Conselho de Gestão do Patrimônio Genético and Instituto Brasileiro do Meio Ambiente (number 003/2008, Protocol 02000.001717/2008-60).

The purity (96%) of uliginosin B was confirmed through HPLC analysis coupled to an ultraviolet detector [1, 22] and its structure was characterized by ^1^H and ^13^C NMR spectra [23]. It was stored at −20°C, protected from light and moisture until use. Immediately before biological testing, it was suspended in saline containing 2% polysorbate 80. Unpublished studies by us demonstrated that, in these storage conditions, ULI has good stability and remains unaltered for approximately 2 years.

### 2.2. Animals

Adult male CF1 mice (25–35 g) were used for* in vivo* and* ex vivo* experiments. Animals were housed under a 12-hour light/dark cycle (lights on at 7:00 am) at constant temperature (23 ± 1°C) with free access to standard certified rodent diet and tap water. All experiments were approved by a local Ethics Committee of Animal Use (UFRGS: 21060/2011) and were in compliance with Brazilian law [24–26] and conformed to the Laboratory Guide for the Care and Use of Animals [27]. Animal handling and all experiments were performed in accordance with international guidelines for animal welfare and measures were taken to minimize animal pain and discomfort.

### 2.3. Behavioral Experiments

Pain sensitivity was assessed by the hot-plate test as described elsewhere [3]. First, each animal freely explored the nonfunctioning hot-plate apparatus for 60 s. Then, the animal returned to its home-cage and the apparatus was turned on and stabilized at 55 ± 1°C. Mice baseline responsiveness was determined by recording the time elapsed until the animal licked one of its hind paws or jumped. Mice that presented a baseline reaction of more than 20 s were not used. Immediately, the animals received one of the following compounds: adenosine A_1_ receptor antagonist: 8-cyclopentyl-1,3-dipropylxanthine (DPCPX) 0.1 mg/kg (0.01 mg/mL, i.p.); adenosine A_2A_-receptors antagonists: 4-(2-(7-amino-2-(furan-2-yl)-[1,2,4]triazolo[1,5-a][1,3,5]triazin-5-ylamino)ethyl)phenol (ZM 241385) 3 mg/kg (0.3 mg/mL, i.p.); inhibitor of adenosine deaminase: erythro-9-(2-hydroxy-3-nonyl) adenine (EHNA) 5 mg/kg (0.5 mg/mL, i.p.); adenosine reuptake inhibitor: dipyridamole (DIP) 30 mg/kg (3 mg/mL, i.p.); ecto-5′-nucleotidase inhibitor: alpha-beta-methylene adenosine 5′-diphosphate (AMPCP) 2 mg/kg (0.2 mg/mL, i.p.). The doses of each tested drug were chosen based on the literature, and lack of antinociceptive effect in the hot-plate test was confirmed in our laboratory [28–31]. After 15 min, the animals were treated with ULI 15 mg/kg (1.5 mg/mL, i.p.) or vehicle (saline plus 2% polysorbate 80; 1 mL/100 g, i.p.) and reexposed to the hot-plate (55 ± 1°C) 30 min later. A maximum latency time of 40 s was imposed (cut-off). The results are expressed as percentages of maximal possible analgesic effect (% MPE) using the following formula:

(1) %  MPE=post-drug latency−pre-drug latencycut-off latency−pre-drug latency×100.

### 2.4. NTPDase and Ecto-5′-nucleotidase Activity

#### 2.4.1. Synaptosomal Preparation

The mice were divided into three groups: handled only (sham), treated with 15 mg/kg ULI (1.5 mg/mL, i.p.), or vehicle (saline plus 2% polysorbate 80, i.p.). After 30 min the animals were killed and the spinal cord and cerebral cortex were removed. The tissues were prepared according to Rozisky et al. [32] and synaptosomes were isolated as described by Nagy and Delgado-Escueta [33]. Protein concentration was determined by the Coomassie blue method [34] using bovine serum albumin as a standard.

#### 2.4.2. Determination of NTPDases and Ecto-5′-nucleotidase Activity

The ATP, ADP, and AMP hydrolysis was performed as described previously [32, 35]. The synaptosomal fraction (10–20 *μ*g protein) was preincubated for 10 min at 37°C in 100 *μ*L of incubation medium containing 45 mM Tris-HCl buffer (pH 8), 0.1 mM EDTA, 1.5 mM CaCl_2_, 5 mM KCl, 10 mM glucose, and 225 mM sucrose for ATP and ADP hydrolysis. For AMP hydrolysis the samples were incubated in 80 *μ*L ecto-5′-nucleotidase incubation medium containing 0.1 M Tris-HCl (pH 7), 10 mM MgCl_2_, and 0.15 M sucrose. The reactions were initiated by the addition of 1 mM ATP, ADP, or AMP and stopped by the addition of 200 *μ*L 10% trichloroacetic acid. Finally, 100 *μ*L samples were taken for the assay of released inorganic phosphate (Pi) [36]. The enzyme activities were expressed as nmol of inorganic phosphate released per minute per milligram of protein (nmol Pi·min^−1^·mg^−1^ protein).

### 2.5. Statistical Analysis

The results were evaluated by one-way analysis of variance (ANOVA) followed by the Student-Newman-Keuls test using the Sigma Stat software, version 2.03 (Jandel Scientific Corporation). All results were expressed as mean ± standard error of the mean (SEM).

## 3. Results

**Figure 2:**
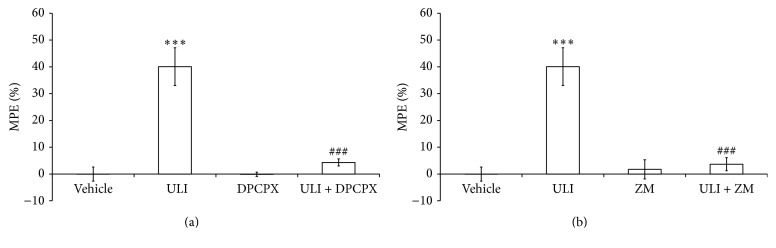
Effect of pretreatment with DPCPX (0.1 mg/kg, i.p. (a)) or ZM 241385 (ZM: 3 mg/kg, i.p. (b)) 15 min before the treatment with uliginosin B (ULI: 15 mg/kg, i.p.) in the hot-plate test. Percentages of maximal possible analgesic effect (% MPE) are presented as means ± SEM (*n* = 9 mice/group). ^*∗∗∗*^ *p* < 0.001 compared to vehicle group; ^###^ *p* < 0.001 compared to uliginosin B (15 mg/kg, i.p.) group (ANOVA followed by Student-Newman-Keuls). Data are presented in mean ± SEM (*n* = 10 mice/group). Significantly different values were detected by one-way ANOVA followed by Student-Newman-Keuls: ^*∗∗∗*^ *p* < 0.001 compared to vehicle.

The influence of selective A_1_ and A_2A_ receptor antagonist pretreatments on the ULI effect in the hot-plate test is depicted in Figure 2. One-way ANOVA revealed a significant antinociceptive effect of ULI (Figure 2(a):  *F*_(3,35)_ = 25.611, *p* < 0.001; Figure 2(b):  *F*_(3,35)_ = 19.555, *p* < 0.001), which was prevented by pretreatment with the adenosine A_1_ receptor antagonist DPCPX (*p* < 0.001) and the adenosine A_2A_ receptor antagonist ZM 241385 (*p* < 0.001).

**Figure 3:**
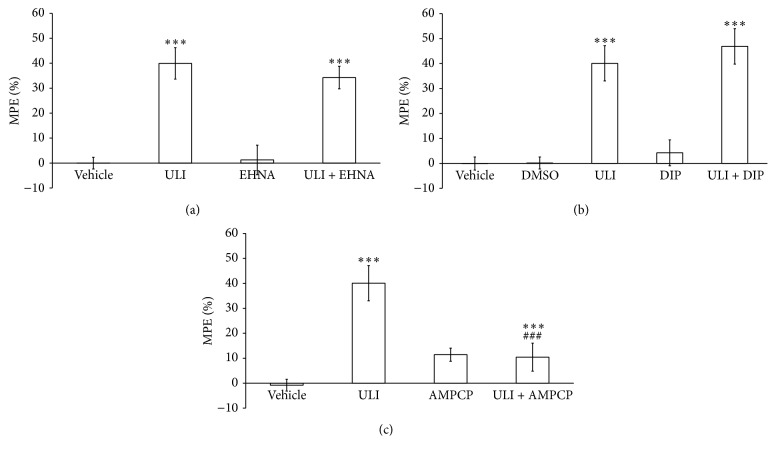
Effect of pretreatment with EHNA (5 mg/kg, i.p. (a)), dipyridamole (DIP: 30 mg/kg, i.p. (b)), or AMPCP (2 mg/kg, i.p. (c)), before treatment with uliginosin B (ULI: 15 mg/kg, i.p.) in the hot-plate test. Percentages of maximal possible analgesic effect (% MPE) are presented as means ± SEM (*n* = 9-10 mice/group). ^*∗∗∗*^ *p* < 0.001 compared to vehicle group; ^###^ *p* < 0.001 compared to uliginosin B (15 mg/kg, i.p.) group (ANOVA followed by Student-Newman-Keuls). Data are presented in mean ± SEM (*n* = 10 mice/group). Significantly different values were detected by one-way ANOVA followed by Student-Newman-Keuls: ^*∗∗∗*^ *p* < 0.001 compared to vehicle.

The data depicted in Figure 3 show the effect of adenosine metabolism on the antinociceptive effect of ULI. Pretreatment with inhibitor of adenosine deaminase (EHNA) or nucleoside transporter inhibitor (dipyridamole) did not affect the ULI nociceptive response. One-way ANOVA revealed a significant effect in the group treated with ULI and ULI plus EHNA or ULI plus dipyridamole in relation to the control groups (Figure 3(a):  *F*_(3,39)_ = 17.819, *p* < 0.001; Figure 3(b):  *F*_(4,44)_ = 19.248, *p* < 0.001). Pretreatment with ecto-5′-nucleotidase inhibitor (AMPCP) prevented the ULI antinociceptive effect on the hot-plate test. One-way ANOVA revealed a significant effect only in the group treated with ULI (Figure 3(c): *F*_(3,35)_ = 12.981, *p* < 0.001), which was prevented by the AMPCP pretreatment (*p* < 0.001).

**Figure 4:**
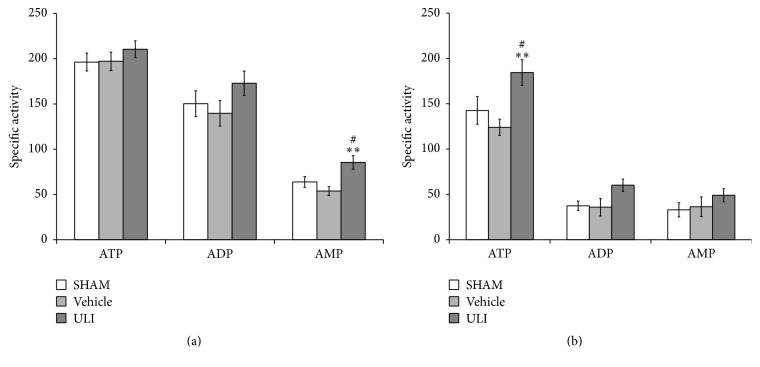
ATP, ADP, and AMP hydrolysis in synaptosomes from spinal cord (a) and cerebral cortex (b) of mice treated with uliginosin B (15 mg/kg, i.p.). Values are presented as means ± SEM (*n* = 5-6 mice/group). Specific enzyme activities were expressed as nmol Pi·min^−1^·mg^−1^ protein. ^*∗∗*^ *p* < 0.01 compared to correspondent sham group; ^#^ *p* < 0.05 compared to correspondent vehicle group (ANOVA followed by Student-Newman-Keuls).

The activities of NTPDases and ecto-5′-nucleotidase were assessed* ex vivo* after acute treatment of mice with ULI (15 mg/kg, i.p.) (Figure 4). In spinal cord synaptosomal preparations, the results showed that the treatment with ULI increased AMP hydrolysis only; ATP and ADP hydrolysis remained unaltered (Figure 3(a), ATP: *F*_(2,17)_ = 0.663, *p* = 0.530; ADP: *F*_(2,17)_ = 1.494, *p* = 0.256; AMP: *F*_(2,17)_ = 6.921, *p* < 0.01). In cerebral cortex synaptosomes, treatment with ULI increased the ATP hydrolysis and there were no changes on ADP and AMP hydrolysis (Figure 3(b), ATP: *F*_(2,14)_ = 5.579, *p* < 0.05; ADP: *F*_(2,14)_ = 3.327, *p* = 0.071; AMP: *F*_(2,14)_ = 0.934, *p* = 0.420).

## 4. Discussion

Herein we demonstrated for the first time the involvement of the purinergic system in the antinociceptive effect of uliginosin B (ULI), a dimeric acylphloroglucinol from* Hypericum* species native to South America. Previous data showed that ULI (15 mg/kg, i.p.) produces antinociceptive effect in hot-plate test [3, 5, 6]. We now show that the pretreatment with DPCPX and ZM 241385, selective adenosine A_1_ and A_2A_ receptor antagonists, respectively, completely prevented the antinociceptive effect of ULI in the mice hot-plate test. This finding indicates that the activation of these receptors mediates the effect of ULI. However, ULI does not have the classical structural requirements for binding to adenosine receptors, since it lacks nitrogen atoms and amine groups, which seem to be crucial for ligands of these receptors [37–40]. Another possibility could be due to allosteric interactions. Nevertheless, although the structure activity relationship is still not completely established, the main allosteric modulators of adenosine receptors also contain nitrogen atoms or amine groups. In addition, compounds that act allosterically and/or orthosterically at the A_1_ adenosine receptor have often close structural resemblance, which suggests that the allosteric site on the A_1_ adenosine receptor is closer or very similar to the orthosteric site of this receptor [41]. Thus, we supposed that the activation of adenosine receptors following ULI treatment could result from increased adenosine availability.

As already mentioned, previous studies by some of us demonstrated that ULI has antidepressant-like effect by inhibiting synaptosomal monoamines reuptake and possibly enhancing the extracellular monoamine availability [2]. Amitriptyline and desipramine, which are antidepressants that increase the availability of monoamines, displayed antinociceptive effects dependent of adenosine receptors activation [8–10, 42–44]. In addition, antidepressants that increase the extracellular availability of monoamines seem to modulate nucleotide hydrolysis in the central nervous system (CNS), presenting stimulatory or inhibitory effect depending on the treatment duration and brain structure [45–47]. The effects of antidepressants on adenosine system seem to reflect increased availability of adenosine following an effect on transport and not necessarily effects on amine transporters [48]. Therefore, we decided to investigate the effect of ULI on nucleotide hydrolysis and adenosine metabolism in order to observe whether adenosine availability has influence on the antinociceptive properties of this compound.

It is noteworthy that our data indicate that the antinociceptive effect of ULI could be, at least in part, dependent of adenosine availability, since it was prevented by pretreatment with AMPCP, an ecto-5′-nucleotidase inhibitor. This result was confirmed by the* ex vivo* assay in spinal cord synaptosomes which pointed to an increase in AMP hydrolysis induced by ULI. In cerebral cortex synaptosomes, treatment with ULI increased the ATP hydrolysis and there were no changes on ADP and AMP hydrolysis. These different effects on AMP hydrolysis could be due to an increased expression of ecto-5′-nucleotidase in the cerebral cortex [49] or to a different processing of the protein, which has been shown to be present in different isoforms in nerve terminals [50, 51], or instead to an abrogation of the negative allosteric modulation of this enzymatic activity by adenine nucleotides [52]. Spinal cord and cerebral cortex, which have been considered as important antinociceptive pathways, possess a high density of adenosine receptors [11, 53, 54].

The fact that ULI stimulates ATP hydrolysis without altering ADP hydrolysis in the cerebral cortex agrees with other studies. ATP, through activation of P_2X3_ receptors, generally facilitates nociceptive transmission while ADP (via P_2Y_ receptors) may decrease the excitatory effect of ATP [55, 56]. ATP can facilitate nociceptive sensitivity by the activation of both ATP-gated ion channels (P2X receptors) and G protein-coupled (P2Y) receptors contributing to nociceptive signaling in peripheral sensory neurons. On the other hand, Gi-coupled P2Y receptors activation can modulate pain neurotransmission [57].

An extensive review by Cunha [58] has pointed that the activation of adenosine A_1_ and A_2A_ receptors is proven to be associated with the release of monoamines, glutamate, and other neuromodulators in different brain regions. In addition, the release of adenosine may be modulated by activation of these neurotransmitter pathways [11, 59–62], as well as opioids [63–66]. Quarta and coworkers [67] have postulated a circuit under physiological conditions of high adenosine release, where regulation of the activation of adenosine A_1_ and A_2A_ receptors could induce glutamate and dopamine release. In this context, it is possible to hypothesize that an increase in adenosine levels could be responsible for an increase in the availability of monoamines and activation of glutamate and opioid receptors, previously described for ULI [2, 3, 5, 6]. Further studies are needed in order to substantiate this assumption.

As a final point, our results demonstrate that the ULI effects on adenosine metabolism involve mainly the modulation of adenosine levels by ecto-5′-nucleotidase activity, since the nociceptive response of this phloroglucinol derivative was not altered by pretreatment with dipyridamole and EHNA, which are nucleoside transporter and ADA inhibitors, respectively. In addition, considering that ATP plays a key role as a danger signal in the brain [68], the ULI ability to increase ATP hydrolysis, with consequent generation of adenosine, may indicate a neuroprotective effect. On the other hand, the results so far do not rule out the possibility that ULI could be a trigger of ATP release. Further experiments are planned in order to investigate these outcomes.

## 5. Conclusion

In conclusion, the present results indicate that uliginosin B increases the availability of adenosine, via ecto-5′-nucleotidases, with consequent activation of adenosine receptors (particularly A_1_ and A_2A_), which play a role in the antinociceptive effect of this phloroglucinol. These findings opened a new avenue for searching the mode of action of this original neuroactive molecular pattern.
